# Supplementary material for: Molecular insight into the initial hydration of tricalcium aluminate
Source: Nat Commun. 2024 Apr 4;15:2929. doi: 10.1038/s41467-024-47164-0 (PMC10995194; doi:10.1038/s41467-024-47164-0)
Supplement: Supplementary file 1 — Supplementary Information [file 41467_2024_47164_MOESM1_ESM.pdf]

Supplementary Information

Molecular insight into the initial hydration of tricalcium aluminate

Xing Ming [a], Wen Si [b], Qinglu Yu [c], Zhaoyang Sun [c], Guotao Qiu [c], Mingli Cao [b], Yunjian Li [a, *], Zongjin Li [a, *]

Emails: xming@must.edu.mo (X. Ming); saiven@mail.dlut.edu.cn (W. Si); yc27803@um.edu.mo (Q. L. Yu); yc17815@um.edu.mo (Z. Y. Sun); yb97411@connect.um.edu.mo (G. T. Qiu) and minglic@dlut.edu.cn (M. L. Cao)

[*] Corresponding authors: liyunjian@must.edu.mo (Y. J. Li) and zjli@must.edu.mo (Z. J. Li)

[a] Faculty of Innovation Engineering, Macau University of Science and Technology, Avenida Wai Long, Taipa, Macao SAR, China.
[b] School of Civil Engineering, Dalian University of Technology, Dalian, China.
[c] Institute of Applied Physics and Materials Engineering, University of Macau, Avenida da Universidade, Taipa, Macao SAR, China.

Supplementary Note 1: Geometry optimization, interface model construction and SCF calculations

The geometry optimization of tricalcium aluminate (C_3A) bulk was carried out using the Vienna Ab initio Simulation Package (VASP)[1,2]. Electron-ion interactions were described using the projector augmented wave (PAW) method[3]. The exchange-correlation potential was determined using the generalized gradient approximation (GGA) with the Perdew-Burke-Ernzerhof (PBE) exchange and correlation functional[4,5]. Valence electron configurations for the elements in these calculations were as follows: H $1s^1$, O $2s^2 2p^4$, Al $3s^2 3p^1$, and Ca $3s^2 3p^6 4s^2$. The self-consistent field (SCF) calculation was performed on the final frame of the ab-initio molecular dynamics (AIMD) trajectory to obtain electronic properties such as the charge density difference (Δq) and local density of states (LDOS). The charge density difference (Δq) was calculated

through the following eq. (1) and projected to z axis and xy plane (Figs. 3a and b):

$$\Delta q = q(system) - q(fragment1) - q(fragment2) \quad (1)$$

where $q(system)$, $q(fragment1)$ and $q(fragment2)$ represent the charge densities of the entire system and different fragments, respectively. In Figs. 3a and b, the system refers to the C_3A/water interface, while the fragments are the C_3A surface and water, respectively. In Supplementary Figs. 10a and b, the system remains the C_3A/water interface, but the fragments are the interface model without the dissolved Ca ion and only the dissolved Ca ion, respectively. Additional calculation details, including energy cutoff, cell size, convergence tolerances for energy and force, and the **k**-point mesh, are provided in Supplementary Table 1. The optimized lattice parameters closely match experimental and other calculation results[6-8], confirming the validity and reproducibility of our calculations. Subsequently, the interface model was constructed based on the optimized bulk C_3A model for subsequent AIMD and well-tempered metadynamics (WT-MetaD) simulations, with further details outlined in Supplementary Fig. 1. During the ~41 ps AIMD sampling, temperature and potential energy evolution profiles are presented in Supplementary Fig. 2. The last ~21 ps of the trajectory was utilized for statistical analysis of the distribution and dynamics of surface ions and water molecules.

Supplementary Table 1. Calculation details of geometry optimization and SCF.

Serials	Geometry optimization	SCF calculations
Surface size	$p(1 \times 1)$	$p(1 \times 1)$
Energy cutoff (eV)	500	500
Energy tolerance (eV/atom)	1×10^{-5}	1×10^{-5}
Force tolerance (eV/Å)	0.01	0.01
**k**-point mesh	5 × 5 × 5	2 × 2 × 1
Optimized lattice constants (a × b × c, $Å^3$)	15.39 ×15.39 ×15.39	15.39 ×15.39 ×52.13
Optimized angels (α, β, γ)	90°, 90°, 90°	90°, 90°, 90°

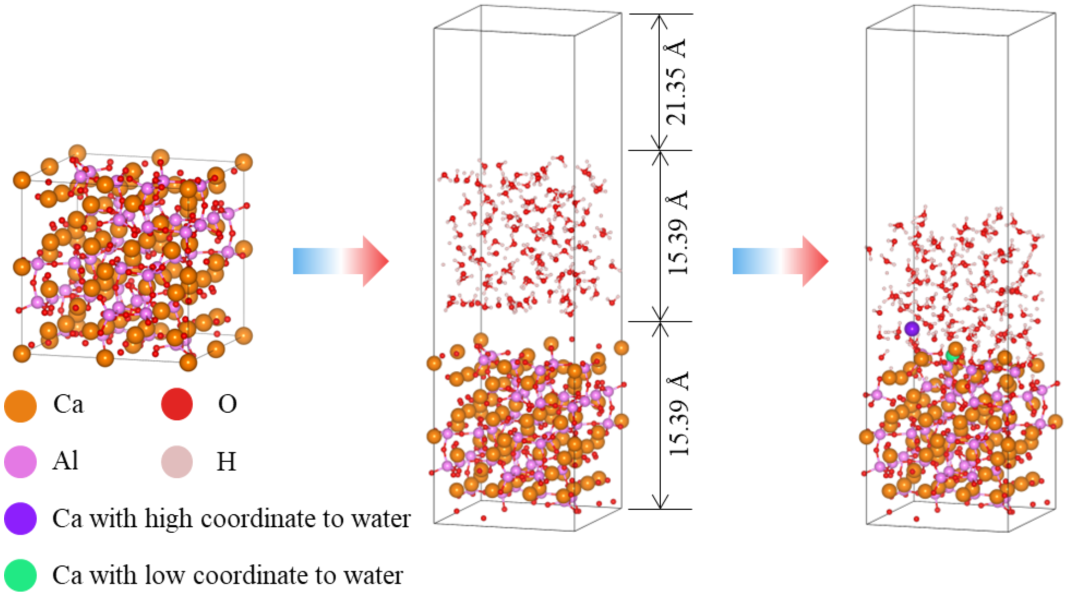

Supplementary Fig. 1. Interface model construction for AIMD and WT-MetaD simulations. The purple Ca ion is used for WT-MetaD simulation on the high water-coordinated Ca ion in the below supplementary materials, and the green one is used for WT-MetaD simulation on the low water-coordinated Ca ion in the main content.

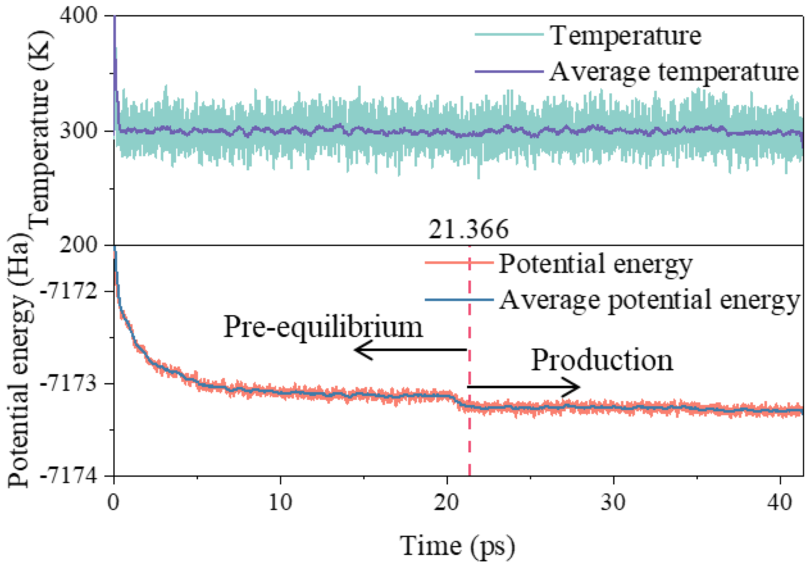

Supplementary Fig. 2. Temperature and potential energy fluctuation versus simulation time.

Supplementary Note 2: Surface energy calculation

We also conducted surface energy calculations to identify the specific cleaved surface for constructing the interface model. Given the symmetry of the cubic system,

only (001), (011) and (111) surfaces are the considered crystallographic planes. The calculations were carried out using the CP2K/Quickstep package[9] by employing density functional theory (DFT) based on a hybrid Gaussian plane wave (GPW) approach[10], known for its computational efficiency, particularly for large systems. The basis set, pseudopotentials, and convergence criteria were consistent with those used for AIMD simulations. Additionally, only the **Γ** point was utilized for relaxing the C_3A bulk and related surfaces due to the large size of the simulation box. Surface energies were then calculated according to eq. (2)[11,12], and the (001) surface exhibited the lowest energy (Supplementary Table 2). This outcome suggests that this face is more susceptible and likely to be cleaved during the synthetic process of cubic C_3A crystal. Consequently, we selected this surface to construct the subsequent interface model.

$$\gamma_s = \frac{1}{2A}(E_s^{unrelax} - NE_b) + \frac{1}{2A}(E_s^{relax} - E_s^{unrelax}) \quad (2)$$

Where E_s^{relax} and $E_s^{unrelax}$ are energies of relaxed and unrelaxed surfaces. N is the number of formula units of the slab, E_b is the bulk energy per formula unit and A is the surface area.

Supplementary Table 2. Surface energies of different cleaved surfaces.

Serials	Structure	Surface energy (J/m^2)
(001)	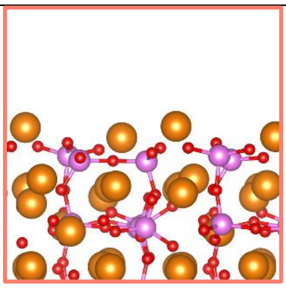	1.91
(011)	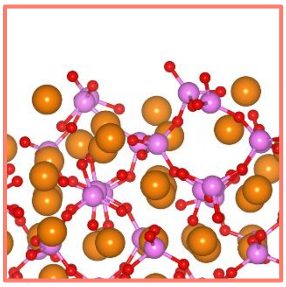	2.23

(111)	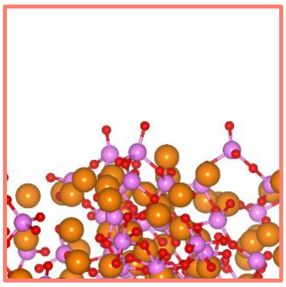	2.03

Supplementary Note 3: Atomic excess ($\Delta\rho$) calculation

To further elucidate the interactions between C_3A surface and water molecules, we performed a specific calculation of atomic excess ($\Delta\rho$) and the density ratio of water molecules (as depicted in Fig. 1c and eq. (3)):

$$\Delta\rho(z) = \frac{2\rho(O_w)_z - \rho(H_w)_z}{2\rho(O_w)_z + \rho(H_w)_z} \tag{3}$$

where $\rho(O_w)$ and $\rho(H_w)$ are number density of O_w and H_w, respectively. Typically, a positive value of atomic excess indicates the accumulation of O_w in a particular region, while conversely, it signifies the localization of H_w in that region[12,13].

Supplementary Note 4: Classical molecular dynamics simulation on C_3A/water interface

A classical molecular dynamics (MD) simulation of the C_3A/water interface was conducted to investigate the initial hydration process of C_3A using a large interface model and an extended simulation duration. The extensive C_3A/water interface was constructed based on the optimized two layers of a *p*(2 × 2) supercell, as previously described. This configuration accommodated a total of 1578 water molecules, achieving an aqueous solution with a density of 1 g/cm^3. Consequently, the supercell dimensions expanded to 30.75 × 30.75 × 87.00 Å^3, encompassing a total of 6846 atoms. The classical MD simulation employed the ReaxFF force field[14,15], integrated into the Large-scale Atomic/Molecular Massively Parallel Simulator (LAMMPS) package[16]. This force field is well-established for exploring interactions between water molecules and cement components[17-19]. We used the optimized parameters for H/O/Ca/Si/Al/S force field[20], which has been successfully used to measure the inhibition effect of

gypsum on C_3A hydration in the presence of aqueous solutions[21]. Periodic boundary conditions (PBC) were applied in all three dimensions. Initially, energy minimization procedures were performed on the interface model using the conjugate gradient algorithm, with energy and force cutoff tolerances set at 1.0×10^{-12} kcal/mol. These energy minimizations aimed to relax both atomic positions and cell parameters. The low and high Taper radius values in the ReaxFF reactive force field were 0.0 and 10.0 respectively, with a charge equilibration precision of 1.0×10^{-6}. To prevent rapid reactions between water and the C_3A surface, the aqueous solutions underwent relaxation for 0.5 ns at 300 K, utilizing the canonical (NVT) ensemble with a temperature damping parameter of 50 fs and a timestep of 0.5 fs. Subsequently, the C_3A surface and water molecules were encountered in the NVT ensemble at a temperature of 300 K for an additional 1 ns to reach equilibrium in potential energy and temperature. This was followed by a 5 ns production run, during which data collection occurred over 20000 frames. The resultant density profile and radial distribution function (RDF) are depicted in Supplementary Figs. 3a and b. Notably, the water molecules exhibited a structured distribution near one side of the surface (as we considered only one side due to the symmetric distribution on both surfaces). The Ca ions exhibited a slight detachment from the surface, resulting in a broader density profile, particularly in the vicinity of the C_3A surface. This observation indicates that dissolution remains a relatively infrequent event, even during the extensive classical MD simulation. Further analysis of the RDF for various ion pairs yielded results highly consistent with those obtained via AIMD simulations, as discussed in the main content. Additionally, a noteworthy observation was made regarding the lower coordination between surface Al and water oxygen ions (O_w), which could not be discerned in our AIMD simulations due to the restricted timescale. This observation suggests a potential dissolution pathway for Al ions initiated by water ligands[22].

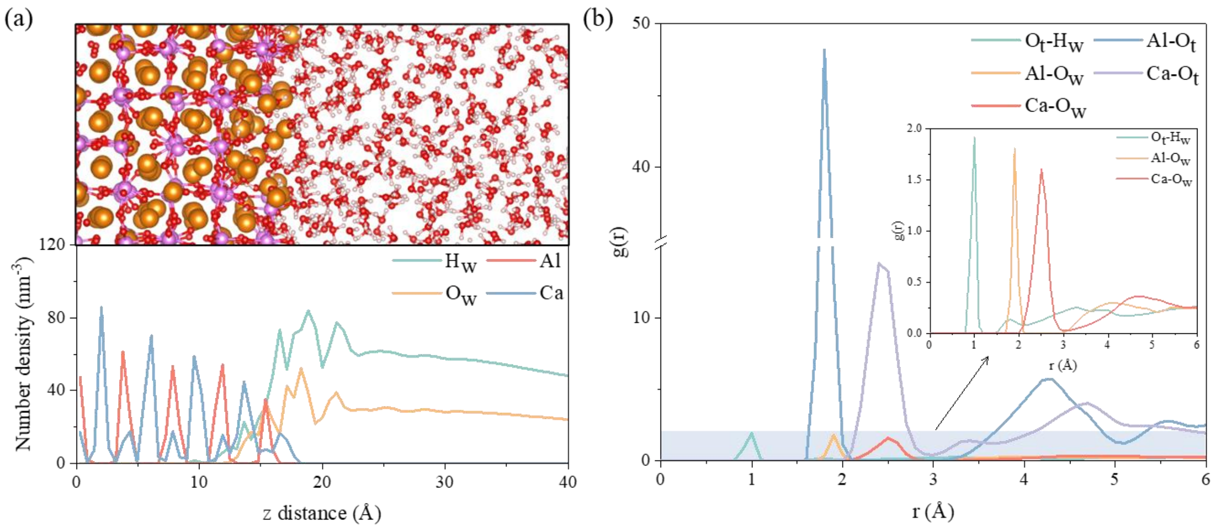

Supplementary Fig. 3. (a) Density profile and (b) radial distribution function (RDF, g(r)) calculated by classical MD simulation.

Supplementary Note 5: Time-dependent density profile

The time-dependent density (TDD) profiles of various surface ions and water molecules were also computed using a methodology akin to that described in the main text (Supplementary Fig. 4). During the simulation, water molecules (H_w and O_w) exhibited a propensity to approach the C_3A surface and rapidly establish coordination with surface O_t and Ca ions. In contrast, the Al ions within the AlO_4 tetrahedron exhibited stability throughout the entire simulation duration, owing to the inherent thermodynamic stability of AlO_4 tetrahedral six-membered rings. This coordinated process facilitated the gradual diffusion of Ca ions from the surface, resulting in a broader distribution in later stages of the simulation. This observation aligns well with the dynamic behavior of surface ions and water molecules discussed in the main content.

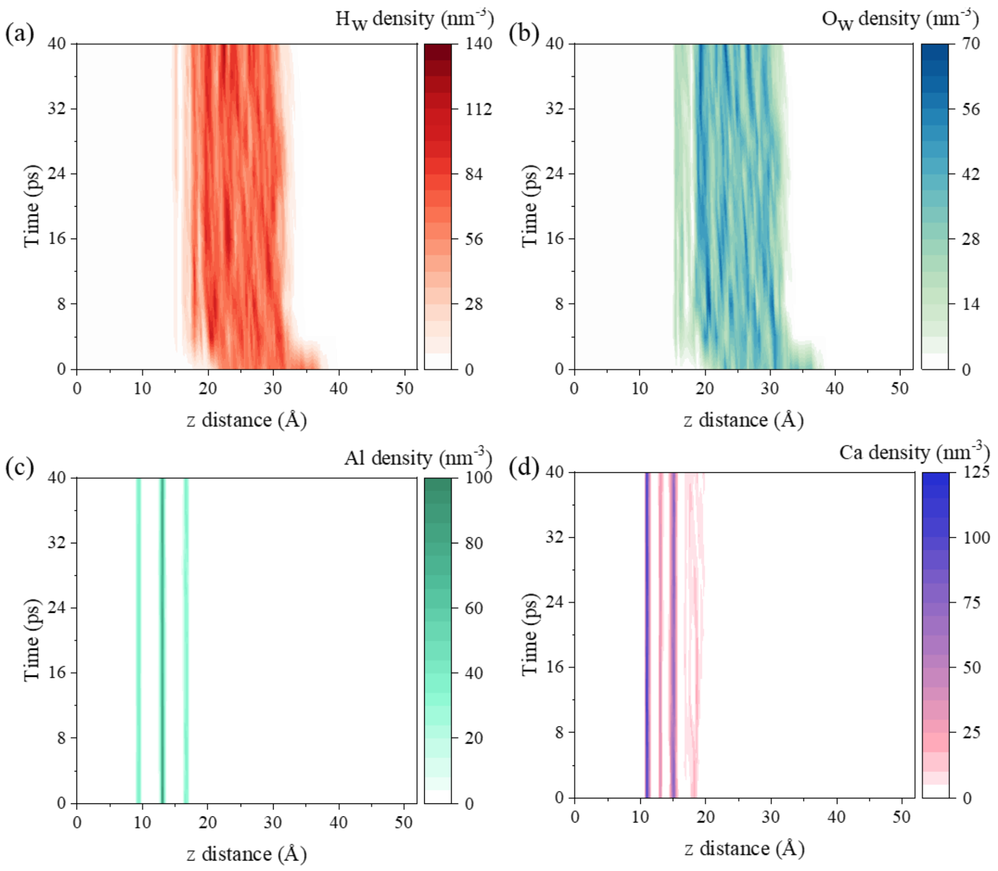

Supplementary Fig. 4. Time-dependent density (TDD) profiles of (a) H_w, (b) O_w, (c) Al and (d) Ca. The TDD profiles are computed by averaging density profiles over 1 ps trajectories sampled every 4 ps.

Supplementary Note 6: Theoretical basis and convergency evolution of WT-MetaD simulations.

In a typical metadynamics simulation, an external history-dependent bias potential is incrementally introduced at specified intervals within the space defined by a set of selected collective variables (CVs). This history-dependent potential is typically represented as a sum of Gaussian functions and can be expressed as follows [22-24]:

$$V\,(\vec{s},t) = \sum_{k\tau<t} W(k\tau)\exp\left(-\sum_{i=1}^{d}\frac{\left[s_i - s_i\big(q(k\tau)\big)\right]^2}{2\sigma_i^2}\right) \qquad (4)$$

where $\tau, W(k\tau), \sigma_i$ are the Gaussian deposition stride, the height and width of the Gaussian functions along the direction of CVs. This approach ensures that the system is steered away from local minima by introducing the metadynamics bias potential,

allowing it to explore the entire phase space and construct the free energy surface. In the conventional metadynamics, the Gaussian height remains constant throughout the simulation. However, we employed the well-tempered variant[25], where the height of the deposited Gaussian diminishes exponentially as the bias accumulates. This adjustment is made to facilitate the convergence of the estimated free energy[26,27].

$$W(k\tau) = W_0 \exp\left(-\frac{V\left(\vec{s}\left(q(k\tau)\right), k\tau\right)}{k_B \Delta T}\right) \tag{5}$$

Where W_0 is the initial Gaussian height and k_B is the Boltzmann constant. ΔT is an input parameter that controls the delay of the Gaussian height. In the asymptotic limit, the bias potential eventually converges and exhibits the following relationship with the underlying free energy:

$$V\ (\vec{s}, t \to \infty) = -\gamma F\ (\vec{s}) + C \tag{6}$$

$$\gamma = \frac{T + \Delta T}{T} \tag{7}$$

where $F\ (\vec{s})$ is the free energy as a function of the selected CVs, C is a constant and γ is the bias factor involved in the input file of PLUMED[28,29].

Metadynamics techniques have proven successful in elucidating atomistic interactions at interfaces, including solid/liquid, liquid/liquid, and liquid/vacuum interfaces. These interfaces play pivotal roles in controlling molecular reactions in diverse fields such as catalysis, geochemistry, and environmental science[22,24,30-36]. The demonstrated success and established foundation in these applications have paved the way for their utilization in simulations of the C_3A/water interface, aimed at revealing the pathways of Ca dissolution. The convergence tests for all the WT-MetaD simulations are presented in Supplementary Figs. 5-7. Over the limited simulation time scale, free energies along individual CV and the free energy differences between two minima exhibit smooth convergence, affirming the reliability and accuracy of our WT-MetaD simulations in probing dissolution pathways, reaction coordinates, and free energy surfaces (FES). Additionally, the simulation durations for each project are detailed in Supplementary Table 3.

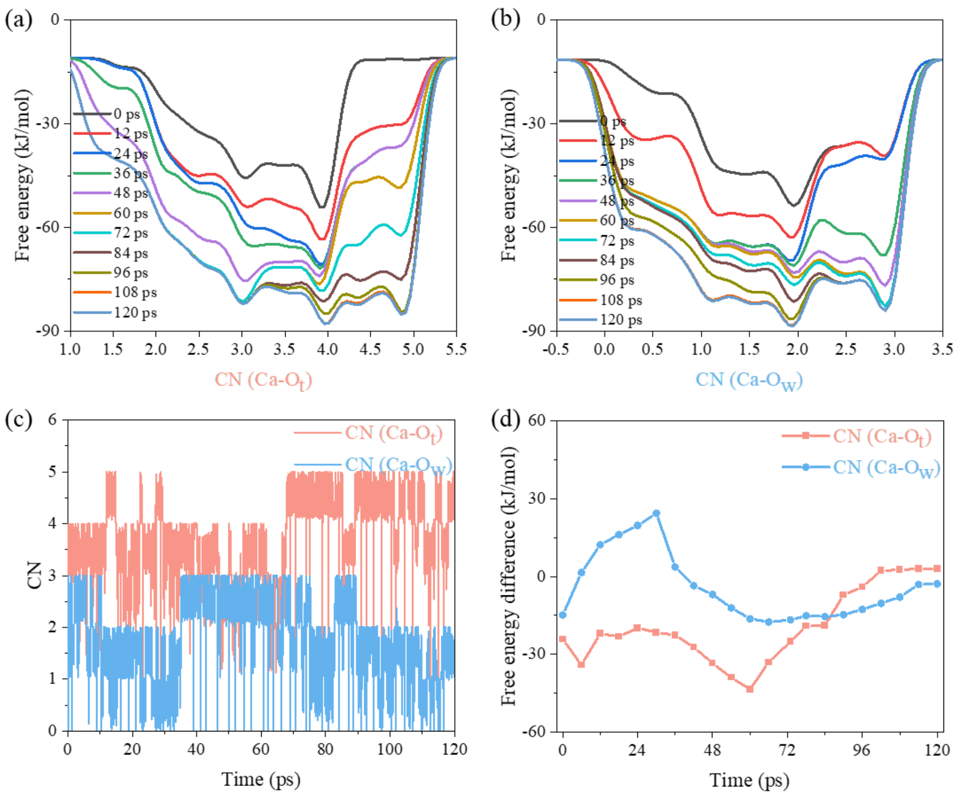

Supplementary Fig. 5. Convergency evolution of WT-MetaD simulation on calcium dissolution with coordination numbers to O_w (CN (Ca-O_w)) ranging from 1 to 3 (low coordination to water molecules). Free energy surfaces as a function of coordination numbers of Ca ions to (a) O_t ions (CN (Ca-O_t)) and (b) O_w ions every 12 ps (600 Gaussian kernels deposited) during the whole simulation time. (c) Time evolution of CN (Ca-O_t) and CN (Ca-O_w). (d) Free energy differences between two basins along the reaction coordinates CN (Ca-O_t) and CN (Ca-O_w) as a function of simulation time.

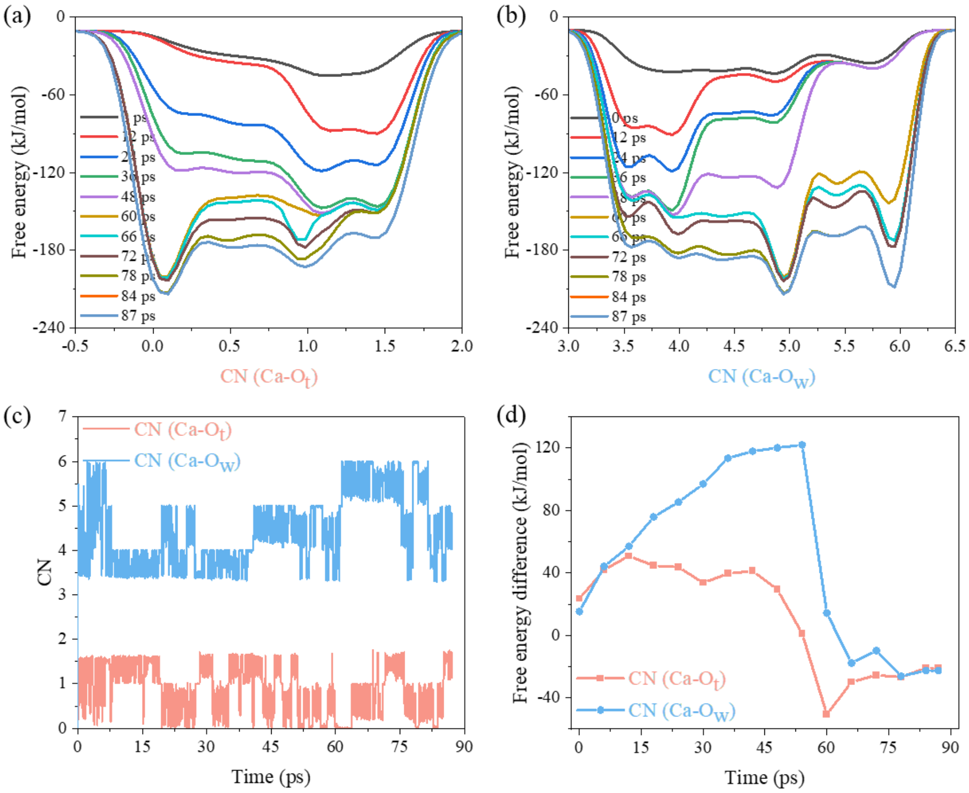

Supplementary Fig. 6. Convergency evolution of WT-MetaD simulation on calcium dissolution with coordination numbers to O_w (CN (Ca-O_w)) ranging from 4 to 6 (low coordination to water molecules). Free energy surfaces as a function of coordination numbers of Ca ions to (a) O_t ions (CN (Ca-O_t)) and (b) O_w ions every 6 ps (300 Gaussian kernels deposited) during the last 27 ps simulation time. (c) Time evolution of CN (Ca-O_t) and CN (Ca-O_w). (d) Free energy differences between two basins along the reaction coordinates CN (Ca-O_t) and CN (Ca-O_w) as a function of simulation time.

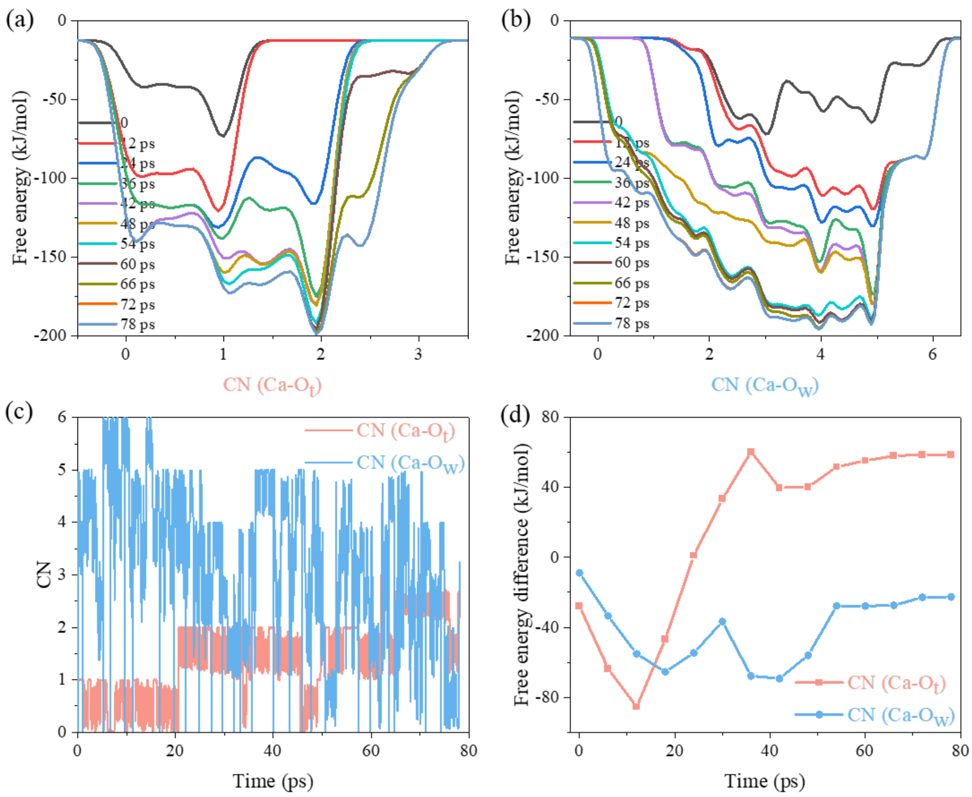

Supplementary Fig. 7. Convergency evolution of WT-MetaD simulation on calcium dissolution with high coordination numbers to O_w (CN (Ca-O_w)). Free energy surfaces as a function of coordination numbers of Ca ions to (a) O_t ions (CN (Ca-O_t)) and (b) O_w ions every 6 ps (300 Gaussian kernels deposited) during the last 42 ps simulation time. (c) Time evolution of CN (Ca-O_t) and CN (Ca-O_w). (d) Free energy differences between two basins along the reaction coordinates CN (Ca-O_t) and CN (Ca-O_w) as a function of simulation time.

Supplementary Table 3. Simulation details for all AIMD and WT-MetaD simulations.

Simulation project	Timestep (fs)	Simulation time (ps)	Elapsed time
AIMD simulation on C_3A/water interface	1	41.38	
Ca dissolution with CN (Ca-O_w) from 1 to 3 (low coordination to water molecules)	1	120.33	
Ca dissolution with CN (Ca-O_w) from 4 to 6 (low coordination to water molecules)	1	87.13	~ 2000 steps/day with 64 CPUs
Ca dissolution with high CN (Ca-O_w)	1	78.09	
Equilibrium AIMD simulation for state E	0.5	8.36	

Supplementary Note 7: WT-MetaD simulation on Ca ions with high CN (Ca-O_w)

To elucidate the influence of the coordination environment on the dissolution pathways of Ca ions from the C_3A surface, we conducted a WT-MetaD simulation on Ca ions exhibiting higher coordination numbers to water molecules than those discussed in the main content (Supplementary Fig. 8). Notably, the dissolution pathways of such Ca ions still adhere to the ligand-exchange mechanism, where water molecules or hydroxy groups engage with the central Ca ions through nucleophilic attacks, ultimately leading to the cleavage of Ca-O_t bonds. However, it is worth noting that the free energies along these reaction pathways exhibit variations. This additional analysis provides further confirmation of the heterogeneous nature of Ca ions dissolving from the C_3A surface.

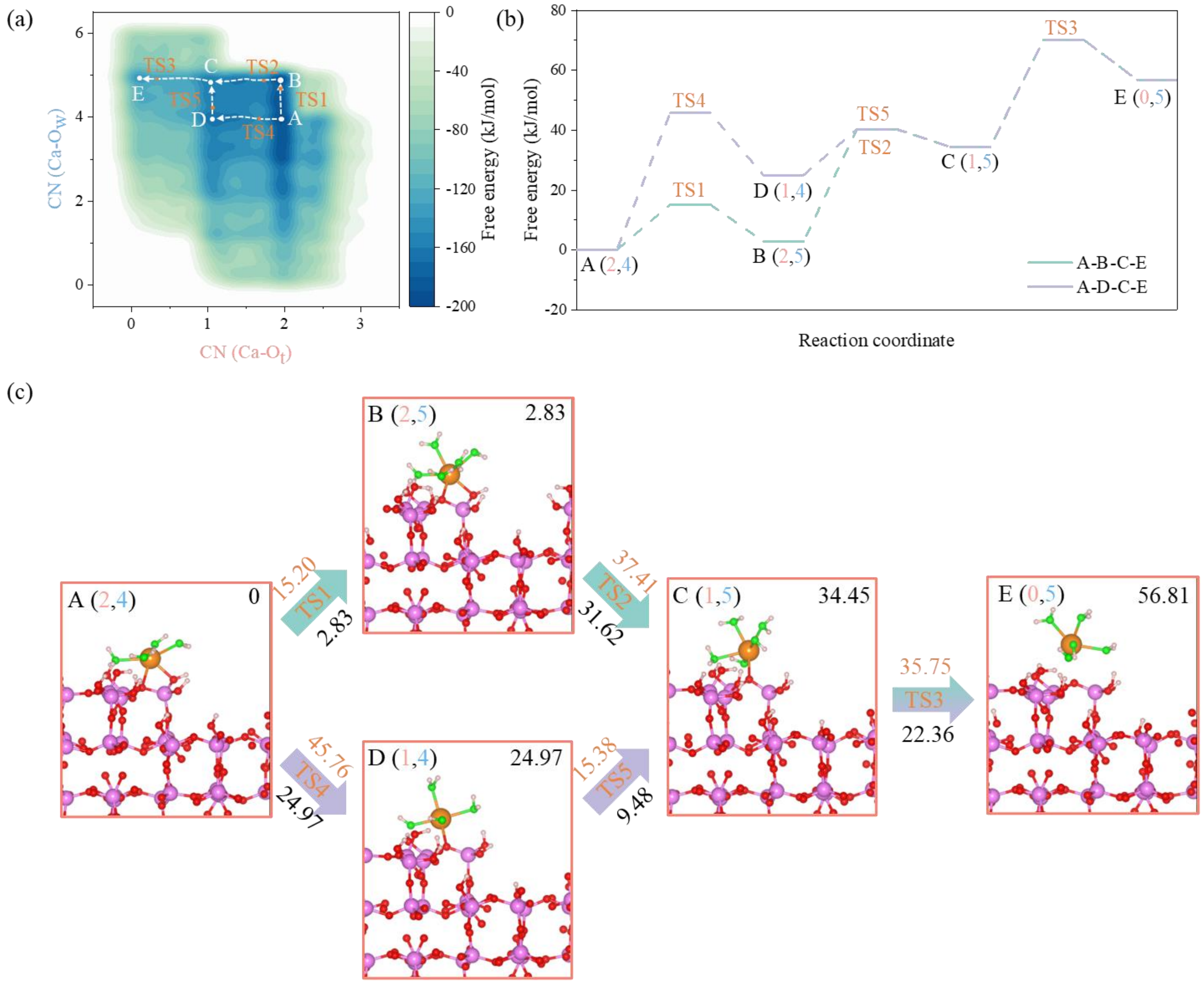

Supplementary Fig. 8. Calcium dissolution with high coordination numbers to water molecules (CN (Ca-O_w)). (a) Free energy surface, (b) reaction coordinate and (c) corresponding snapshots of configuration evolution along the reaction pathways. In the above representations, the sates along the dissolution pathways are notated in the form of X (CN (Ca-O_t), CN (Ca-O_w)), where X indicates the state number on the free energy surface or the snapshots (e.g., the A, B, etc.), CN (Ca-O_t) and CN (Ca-O_w) represent coordination numbers of Ca to O_t and O_w, respectively in state X. "TS" denotes the transition state. The upper right corners display the states with Helmholtz free energy values (in kJ/mol) relative to state A. The saffron yellow values above the arrows represent free energy barriers (in kJ/mol), while the black values below the arrows denote the free energy differences between two adjacent states (in kJ/mol). The arrows with varied colors signify the distinct reaction pathways that align with those depicted in Supplementary Fig. 8b.

Supplementary Note 8: Configuration evolution of state E with and without sulfate ions

The local minima state E identified on the FES through WT-MetaD simulations underwent subsequent equilibration in AIMD simulations (Supplementary Fig. 9). The simulation parameters remained consistent with those previously described, except for a timestep of 0.5 fs using the original proton mass. An intriguing observation was made, wherein the state E (0,6) rapidly transitioned to state I (0,5), and subsequently to either G (1,6) or F (1,5). This phenomenon can be attributed to the pronounced affinity between surface Ot ions and central Ca ions.

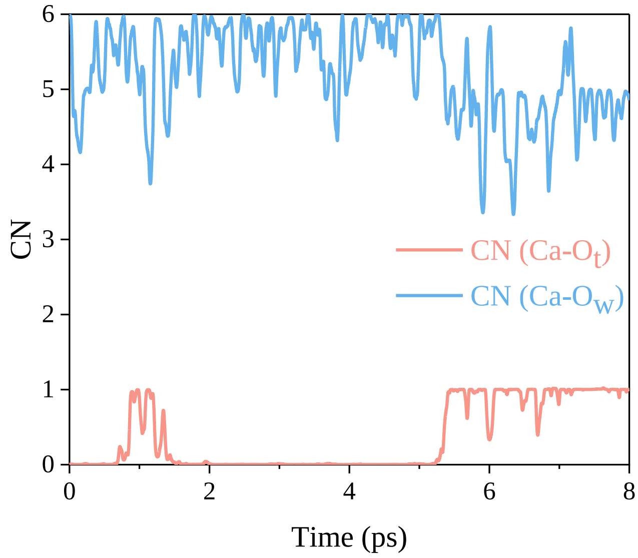

Supplementary Fig. 9. Transformation of state E (0,6) to other states during the equilibrium AIMD simulation.

Supplementary Note 9: Interfacial charge distribution around the dissolved Ca ion

The interfacial charge distribution around the dissolved Ca ion was computed to illustrate the positively charged nature of the remaining Al-rich layer. The calculation details are provided in Supplementary Note 1, and the results can be observed in Supplementary Fig. 10. A charge depletion area is evident around the dissolved Ca ion, signifying its positive charge. Consequently, the partially dissolved C_3A surface can demonstrate a positive zeta potential and electrostatically attract the available sulfate

ions in the vicinity areas.

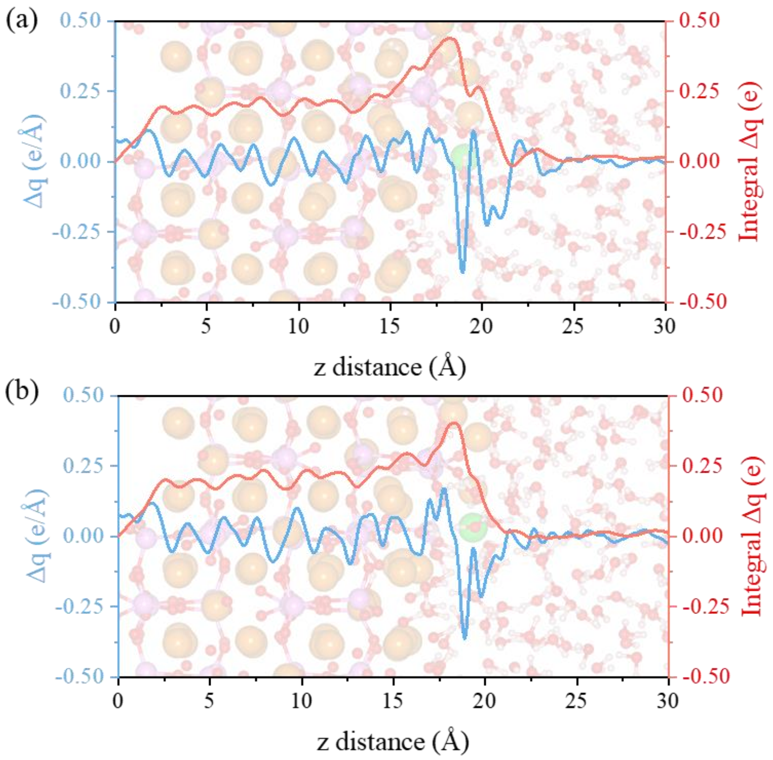

Supplementary Fig. 10. Interfacial charge distribution around the dissolved Ca ion for (a) sate F (1,5) and (b) state G (1,6). The positive Δq value refer charge accumulation regions around these ions, while a negative value means charge depletion regions.

Supplementary Note 10: Dynamics of AlO_4 tetrahedra during AIMD simulation

The configuration evolution of AlO_4 tetrahedra during AIMD simulation can be seen in the attached movie (Supplementary Movie 1.mp4).

Supplementary References

1. Kresse, G., Furthmuller, J. Efficient iterative schemes for ab initio total-energy calculations using a plane-wave basis set. *Physical Review B* **54**, 11169-11186 (1996).

2. Kresse, G., Hafner, J. Ab initio molecular-dynamics simulation of the liquid-

metal-amorphous-semiconductor transition in germanium. *Phys Rev B Condens Matter* **49**, 14251-14269 (1994).

3. Blöchl, P. E. Projector augmented-wave method. *Physical Review B* **50**, 17953-17979 (1994).

4. Perdew, J. P., Burke, K., Ernzerhof, M. Generalized gradient approximation made simple. *Phys Rev Lett* **77**, 3865-3868 (1996).

5. Perdew, J. P*., et al.* Atoms, molecules, solids, and surfaces_ applications of the generalized gradient approximation for exchange and correlation. *Phys Rev B Condens Matter* **46**, 6671-6687 (1992).

6. Manzano, H., Dolado, J. S., Ayuela, A. Structural, mechanical, and reactivity properties of tricalcium aluminate using first-principles calculations. *Journal of the American Ceramic Society* **92**, 897-902 (2009).

7. Mishra, R. K., Fernandez-Carrasco, L., Flatt, R. J., Heinz, H. A force field for tricalcium aluminate to characterize surface properties, initial hydration, and organically modified interfaces in atomic resolution. *Dalton Trans* **43**, 10602-10616 (2014).

8. Mondal, P., Jeffery, J. W. The crystal structure of tricalcium aluminate, ca3al2o6. *Acta Cryst* **B31**, 689-697 (1975).

9. Vandevondele, J., Krack, M., Mohamed, F., Parrinello, M., Chassaing, T., Hutter, J. Quickstep: Fast and accurate density functional calculations using a mixed gaussian and plane waves approach. *Computer Physics Communications* **167**, 103-128 (2005).

10. Lippert, B. G., Parrinello, J. H., Michele. A hybrid gaussian and plane wave density functional scheme. *Molecular Physics* **92**, 477-488 (2010).

11. Li, Q., Rellán-Piñeiro, M., Almora-Barrios, N., Garcia-Ratés, M., Remediakis, I. N., López, N. Shape control in concave metal nanoparticles by etching. *Nanoscale* **9**, 13089-13094 (2017).

12. Li, Y., Ai, H., Lo, K. H., Kong, Y., Pan, H., Zongjin Li. Insight into adsorption mechanism of water on tricalcium silicate from first-principles calculations. *Cement and Concrete Research* **152**, 106684 (2022).

13. Réocreux, R., Jiang, T., Iannuzzi, M., Michel, C., Sautet, P. Structuration and dynamics of interfacial liquid water at hydrated γ-alumina determined by ab initio molecular simulations: Implications for nanoparticle stability. *ACS*

Applied Nano Materials **1**, 191-199 (2017).

14. Van Duin, A. C. T., Dasgupta, S., Lorant, F., Goddard, W. A. Reaxff: A reactive force field for hydrocarbons. *The Journal of Physical Chemistry A* **105**, 9396-9409 (2001).
15. Van Duin, A. C. T., Strachan, A., Stewman, S., Zhang, Q., Xu, X., Goddard, W. A. Reaxffsio reactive force field for silicon and silicon oxide systems. *The Journal of Physical Chemistry A* **107**, 3803-3811 (2003).
16. Plimpton, S. Fast parallel algorithms for short-range molecular dynamics. *Journal of Computational Physics* **117**, 1-19 (1995).
17. Sun, M., Geng, G., Xin, D., Zou, C. Molecular quantification of the decelerated dissolution of tri-calcium silicate (c3s) due to surface adsorption. *Cement and Concrete Research* **152**, 106682 (2022).
18. Qi, C., Manzano, H., Spagnoli, D., Chen, Q., Fourie, A. Initial hydration process of calcium silicates in portland cement: A comprehensive comparison from molecular dynamics simulations. *Cement and Concrete Research* **149**, 106576 (2021).
19. Manzano, H., Durgun, E., Lopez-Arbeloa, I., Grossman, J. C. Insight on tricalcium silicate hydration and dissolution mechanism from molecular simulations. *ACS Appl Mater Interfaces* **7**, 14726-14733 (2015).
20. Liu, L., Jaramillo-Botero, A., Goddard, W. A., 3rd, Sun, H. Development of a reaxff reactive force field for ettringite and study of its mechanical failure modes from reactive dynamics simulations. *J Phys Chem A* **116**, 3918-3925 (2012).
21. Zhu, J., Shen, D., Wu, W., Jin, B., Wu, S. Hydration inhibition mechanism of gypsum on tricalcium aluminate from reaxff molecular dynamics simulation and quantum chemical calculation. *Molecular Simulation* **47**, 1465-1476 (2021).
22. Reocreux, R., *et al.* Reactivity of shape-controlled crystals and metadynamics simulations locate the weak spots of alumina in water. *Nat Commun* **10**, 3139 (2019).
23. Nair, N. N., Schreiner, E., Marx, D. Glycine at the pyrite-water interface: The role of surface defects. *J Am Chem Soc* **128**, 13815-13826 (2006).
24. Sun, M., Gao, X., Zhang, Z., Zou, C., Xin, D., Geng, G. Stepwise dissolution of silica surface in alkaline solution revealed by molecular modeling. *Journal of the American Ceramic Society*, 1-16 (2023).

25. Polino, D., Parrinello, M. Kinetics of aqueous media reactions via ab initio enhanced molecular dynamics: The case of urea decomposition. *J Phys Chem B* **123**, 6851-6856 (2019).
26. Barducci, A., Bussi, G., Parrinello, M. Well-tempered metadynamics: A smoothly converging and tunable free-energy method. *Phys Rev Lett* **100**, 020603 (2008).
27. Dama, J. F., Parrinello, M., Voth, G. A. Well-tempered metadynamics converges asymptotically. *Phys Rev Lett* **112**, 240602 (2014).
28. Bonomi, M.*, et al.* Plumed: A portable plugin for free-energy calculations with molecular dynamics. *Computer Physics Communications* **180**, 1961-1972 (2009).
29. Tribello, G. A., Bonomi, M., Branduardi, D., Camilloni, C., Bussi, G. Plumed 2: New feathers for an old bird. *Computer Physics Communications* **185**, 604-613 (2014).
30. Chen, Q., Liu, J., Yang, B. Identifying the key steps determining the selectivity of toluene methylation with methanol over hzsm-5. *Nat Commun* **12**, 3725 (2021).
31. Martonak, R., Donadio, D., Oganov, A. R., Parrinello, M. Crystal structure transformations in sio2 from classical and ab initio metadynamics. *Nat Mater* **5**, 623-626 (2006).
32. Grifoni, E., Piccini, G., Lercher, J. A., Glezakou, V. A., Rousseau, R., Parrinello, M. Confinement effects and acid strength in zeolites. *Nat Commun* **12**, 2630 (2021).
33. Stack, A. G., Raiteri, P., Gale, J. D. Accurate rates of the complex mechanisms for growth and dissolution of minerals using a combination of rare-event theories. *J Am Chem Soc* **134**, 11-14 (2012).
34. Biswas, S., Kwon, H., Barsanti, K. C., Myllys, N., Smith, J. N., Wong, B. M. Ab initio metadynamics calculations of dimethylamine for probing pk(b) variations in bulk vs. Surface environments. *Phys Chem Chem Phys* **22**, 26265-26277 (2020).
35. Li, Y., Pan, H., Liu, Q., Ming, X., Li, Z. Ab initio mechanism revealing for tricalcium silicate dissolution. *Nat Commun* **13**, 1253 (2022).
36. Xia, D., Chen, J., Xie, H. B., Zhong, J., Francisco, J. S. Counterintuitive

oxidation of alcohols at air-water interfaces. *J Am Chem Soc* **145**, 4791-4799 (2023).
