# Supplementary material for: Molecular insight into the initial hydration of tricalcium aluminate
Source: Nat Commun. 2024 Apr 4;15:2929. doi: 10.1038/s41467-024-47164-0 (PMC10995194; doi:10.1038/s41467-024-47164-0)
Supplement: Supplementary file 3 — Description of Additional Supplementary Files [file 41467_2024_47164_MOESM3_ESM.pdf]

Description of Additional Supplementary Files:

Supplementary Movie 1: Dynamics of AlO_4 tetrahedra during AIMD simulation
